# Prevalence of disability according to multimorbidity and disease clustering: a population-based study

**DOI:** 10.15256/joc.2011.1.3

**Published:** 2011-12-27

**Authors:** Alessandra Marengoni, Sara Angleman, Laura Fratiglioni

**Affiliations:** ^1^Aging Research Centre, NVS Department, Karolinska Institutet and Stockholm University, Stockholm, Sweden; ^2^Stockholm Gerontology Research Centre, Stockholm, Sweden; ^3^Department of Medical and Surgery Sciences, University of Brescia, Brescia, Italy; ^*^These authors contributed equally to this work.

**Keywords:** chronic diseases, disability, multimorbidity, older adults

## Abstract

**Background:**

The prevalence of chronic diseases has increased with population ageing, and research has attempted to elucidate the correlation between chronic diseases and disability. However, most studies in older populations have focused on the effect of single disabling conditions, even though most older adults have more than one chronic disease (multimorbidity).

**Objective:**

The aims of this study were to evaluate the association of disability with disease, in terms of multimorbidity and specified pairs of diseases, in a population-based study of older adults. Materials and Methods: Using the Kungsholmen Project, we estimated the prevalence of disability by the number of chronic diseases, disease status by organ systems, and in specific pairs of chronic conditions, in a Swedish population (*n*=1,099; ≥77 years). Disability was defined as need of assistance in at least one activity of daily living (Katz index).

**Results:**

Functional disability was seen in 17.9% of participants. It increased as the number of chronic diseases increased. The prevalence of disability varied greatly amongst specific pairs of diseases: from 6.7% in persons affected by hypertension and atrial fibrillation to 82.4% in persons affected by dementia and hip fracture. In multivariate logistic regression models, the disease pairs that were significantly associated with the highest increased relative odds of disability contained dementia (dementia–hip fracture, dementia–CVD, and dementia–depression).

**Conclusions:**

Our findings suggest specific pairs of diseases are much more highly associated with disability than others, particularly diseases coupled with dementia. This knowledge may improve prevention of disablement and planning of resource distribution.

Journal of Comorbidity 2011;1:11–18

## Introduction

The demographic phenomenon known as population ageing that is occurring now in developed, as well as in some less-developed countries, has resulted in the increasing prevalence of chronic diseases. This is one of the major challenges of the aging society, and in order to face this challenge, it is important to understand how multimorbidity and specific combinations of chronic diseases affect major outcomes, such as functional disability.

Whilst the presence of disease is viewed as the initial change underlying disability onset in the elderly [1], several researchers have attempted to elucidate the correlation between chronic conditions and disability. However, the majority of studies conducted in older populations have focused on the effect of single disabling conditions, for instance musculoskeletal diseases [2], depression [3], and circulatory diseases [4].

Verbrugge and colleagues were the first to explore the impact of the coexistence of multiple conditions on disability, and demonstrated that with increasing numbers of chronic diseases, disability increased almost exponentially [5]. Another study investigated the effect of comorbidity in arthritis on disability and showed that arthritis and other conditions may act synergistically on functional status [6]. Data from the Women’s Health and Aging Study showed that specific disease pairs, such as arthritis and visual impairments, arthritis and hypertension, heart diseases and cancer, and others were synergistically associated with different types of disability [7]. A previous longitudinal study from the cohort of the Kungsholmen Project showed that an increasing number of chronic conditions was associated with an increasing risk of functional decline over time [8].

## Objectives

The aims of this study were to evaluate the strength of the association of disability with disease, in terms of both multimorbidity, as well as different specific pairs of diseases, in a population-based study of older adults.

## Materials and Methods

### Study population

The Kungsholmen Project is a population-based prospective study on aging and dementia [9], including all inhabitants in the Kungsholmen district of Stockholm, Sweden, who were aged 75 years and older in October 1987. The baseline assessment was carried out between 1987 and 1989 and was followed by four examinations spaced approximately 3 years apart (FU1–4). Each subject was sent a personal letter explaining the study and the importance of participation but stating that it was voluntary and that they could discontinue participation at any time. The Kungsholmen Project was approved by the ethics committee of the Karolinska Institute.

Of the 1,700 elderly persons who agreed to participate in the baseline examination (1987–1989), 429 died and 172 moved or refused to participate at the first follow-up (1991–1993). In the present study, the population (*n*=1,099) consisted of participants at the first follow-up, at which time they were all examined by physicians. We did not use the Kungsholmen Project baseline assessment as only a subsample of the population underwent clinical examination, and information on the presence of diseases was derived for the whole population from the Stockholm Inpatient Registry, which was judged to be too limited for a study aiming to identify disease clustering. Detailed information about the study design of the Kungsholmen Project has been previously published [9].

### Data collection

All participants were examined following a standardized protocol, including a social interview, a neuropsychological battery, and a medical examination, lasting about 3 hours. The clinical examination included general, neurological, and psychiatric status, as well as medication use. The elderly and their next-of-kin were interviewed by trained nurses using a structured questionnaire on living conditions and social status. Information on the highest educational level achieved was obtained at baseline directly from the subject or from an informant.

### Chronic diseases assessment

A disease was classified as chronic if one or more of the following characteristics were present [10, 11]:

- state of permanence
- caused by non-reversible pathological alteration
- requiring rehabilitation
- requiring a long period of care.

Chronic diseases were diagnosed by the examining physician according to clinical examination, medical history, laboratory data, and current use of medications, or ascertained using the computerized Stockholm inpatient register system, except for the following disorders: (i) sensory function (deafness was defined as being unable to hear the interviewer’s voice, and visual impairment was defined as being blind or almost blind); (ii) dementia, different dementia types, and major depression were diagnosed by a psychiatrist according to the Diagnostic and Statistical Manual of Mental Disorders, Revised Third Edition (DSM-III-R) criteria [12]; (iii) anaemia was defined as haemoglobin <13 g/dL in males and <12 g/dL in females [13]. A total of 30 chronic diseases were selected in the population. In order to study the association of diseases with disability, we analysed participants’ health status using three different methods. First, we calculated the number of diseases affecting each person. All the chronic diseases detected in the population were included in the calculation. The variable was constructed in five categories: no disease, one disease, two diseases, three diseases, and four or more diseases. Second, we used the International Classification of Diseases – Ninth Revision (ICD-9) [14] to group our selected diseases into main organ systems: cardiovascular system diseases (heart diseases and hypertension); cerebrovascular system diseases (stroke and transient ischaemic attack); mental diseases (dementia, depression, and schizophrenia); neurosensory diseases (parkinsonism, epilepsy, deafness, and visual impairments); endocrinological system diseases (diabetes and thyroid problems); musculoskeletal system diseases (hip fracture, arthritis, polymyalgia, and osteoporosis); respiratory system diseases (chronic obstructive pulmonary diseases); malignancy (blood and solid); and blood diseases (anaemia). Furthermore, specific pairs of chronic diseases were identified in order to analyse their different strengths of association with disability. For the purpose of this study, we focused on the most common chronic conditions in which the observed co-prevalence exceeded the expected one, as previously described [15].

### Functional status

Disability was determined from functional status as measured by the Katz index of activities of daily living (ADL) [16], which is a hierarchical scale formed by dependency in the following six activities: bathing, dressing, going to the toilet, transferring, continence, and feeding. Level of dependence was expressed in grades: ‘A’ represented the most independent (requiring no personal assistance in all six activities) and ‘G’ represented the most dependent grade (requiring assistance in all six activities). In this article, functional independence was defined as the need for assistance in none of the activities (A), and disability was defined as need of assistance in one to six specified activities (from B to G). Data on functional status were collected by nurse interviews and observations of the subjects. Data on functional status were missing for 15 subjects, and data on educational level were missing for seven subjects, leaving 1,077 participants available for the present analysis.

### Statistical analysis

Prevalence of disability per 100 persons was calculated by the number of chronic conditions and by main organ systems. Prevalence of disability was calculated, stratified by the pairs of diseases, as identified above. Logistic regression models were run in order to analyse the association of specific pairs of diseases (independent variable, categorized in four levels: having neither of the specified diseases, having the first but not the second disease, having the second but not the first disease, and having both specified diseases) with disability (dependent variable, categorized in two levels: requiring no assistance in any of the activities and requiring assistance in at least one of the activities) after adjustment for age, sex, and education (measured as highest level of education attained and categorized as 8+ versus ≤7 years of schooling). For each specific pair of diseases, the model computed the relative odds of disability amongst participants who had both diseases in the pair relative to participants who did not have either disease in the pair.

Finally, in order to test if specific pairs of diseases had a synergistic effect on disability or if the association was mainly due to a single disease included in the cluster, further logistic regression models were run, each assessing the effect of only one clinical condition.

## Results

### Prevalence of disability

Of the 1,084 participants at baseline, 194 (17.9%) had disability in ADL. Persons with disability were significantly more likely to be older, female, widowed, and living in institutions (Table 1).

### Multimorbidity and disability

Only 15% of the population at baseline was free from chronic diseases. Thirty percent had only one disease, and 55% had multimorbidity (at least two diseases). Table 2 describes the prevalence of disability by increasing number of diseases. Almost all of the participants without any chronic disease were functionally independent. Increasing number of diseases was associated with increasing prevalence of disability (*p* for trend <0.001). Compared with those with no diseases, prevalence was roughly fourfold higher for those with one disease, fivefold higher for those with two or three diseases, and over sixfold higher for those with at least four diseases. Participants with four or more chronic diseases (range 4–7, mean 4.5±0.7) had a prevalence of disability of 28%, a significantly higher prevalence compared with those with only one disease (16.8%, *p*=0.005) or no disease (4.3%, *p*<0.0001).

### Organ systems and disability

The main organ systems detected in our study are described in Table 2. There was wide variation in the prevalence level of types of diseases by organ system. Cardiovascular diseases were by far the most prevalent, affecting over half of the study population (Table 2). The next most prevalent disease groups were the mental disease and neurosensory disease groups; however, these had only half the prevalence of the cardiovascular disease group. Despite these great differences in disease prevalence, the differences in disability prevalence were minor among the majority of organ systems involved, with the exceptions of the mental disease and cerebrovascular disease (CVD) groups. Prevalence of disability ranged from 11.9% in the cardiovascular disease group to 19.4% in the neurosensory disease group, yet it was 42.0% in the CVD group and 50.9% for the mental disease group (Table 2).

### Specific pairs of diseases and disability

**Figure 1:**
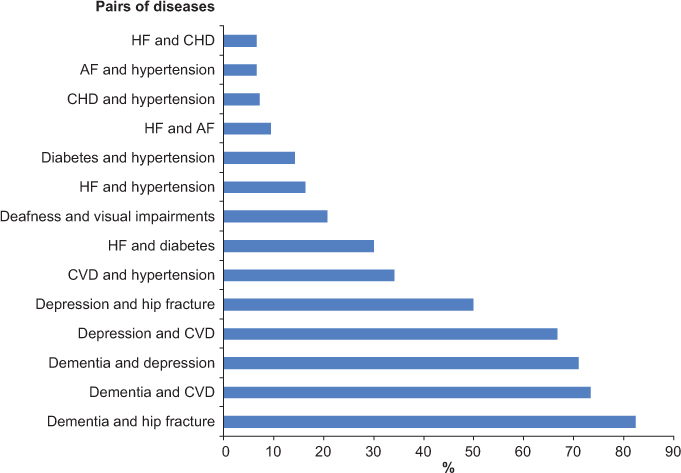
Prevalence of disability in activities of daily living per 100 persons according to specific pairs of chronic diseases. AF, atrial fibrillation; CHD, coronary heart disease; CVD, cerebrovascular disease; HF, heart failure.

Prevalence of disability varied greatly according to specific pairs of diseases; the lowest prevalence was found in persons affected by atrial fibrillation (AF) and hypertension, and by heart failure (HF) and coronary heart disease (CHD) (6.7%), and the highest was in persons affected by dementia and hip fracture (82.4%) (Figure 1). In general, the combination of cardiovascular diseases with each other, or with metabolic diseases was associated with the lowest prevalence of disability, whereas the various combinations of four specific diseases, dementia, depression, CVD, or hip fracture were associated with the highest prevalence of disability.

Findings from multivariate logistic regression models testing the association of specific pairs of diseases with disability, showed that only five out of 14 disease pairs were significantly associated with an increased relative odds of disability after adjustment for age, sex, and education (Table 3). The majority of these pairs consisted of the same combinations of the four specific diseases (dementia, depression, CVD, and hip fracture) associated with the highest prevalence of disability in Table 2, as well as the pair of CVD and hypertension. The odds of the association varied from 2.3 [95% confidence interval (CI) 1.1–4.9] in persons affected by CVD and hypertension to 56.3 (95% CI 15.3–207.8) in those affected by dementia and hip fracture (Table 3). The very high point estimates must be interpreted with caution, as it can be seen from Table 3 that the number was quite small for having both diseases in some of the specific pairs, explaining the very wide CIs. Also, three specific pairs of diseases were found to be significantly associated with a *decreased* relative odds of disability: HF and CHD, AF and hypertension, and CHD and hypertension.

Finally, for those specific pairs of diseases that had a significantly increased relative odds of disability, having both diseases appears to be associated with a much higher risk of disability, compared with having just one of either of the diseases in the pair (Table 4).

## Discussion

In the last few decades, the assessment of disability has become an essential part of the comprehensive evaluation of the health status of the elderly. It has been estimated that 18% of community-dwelling elderly persons report at least one limitation in ADL which is very similar to the prevalence of disability found in the Kungsholmen population; moreover, the frequency of disability rises with age [17]. This study shows that the prevalence of disability in elderly people greatly varies according to the number and type of chronic diseases present. Disability increases as the number of chronic diseases increases. The prevalence of disability is lowest in cardiovascular and highest in mental and cerebrovascular system diseases. There are great variations in the association between specific pairs of conditions and disability, both in terms of prevalence and odds ratios.

In agreement with previous research [5, 18, 19], the results of this study underline the close association between the number of diseases and disability in old age. In fact, persons without any chronic disease were mostly independent of ADL, whereas the prevalence of disability increased up to 28% in those affected by four or more chronic conditions. These findings have been confirmed in prospective studies which showed that multimorbidity has a negative effect on disability onset and worsening during time [8, 20]. However, there were a number of people affected by chronic diseases without disability. Despite the fact that the role of chronic conditions in causing functional impairment is intuitively important, we still lack consensus on the pathway from disease to disability [21]. One hypothesis is that diseases lead to impairments (anatomic and structural), which lead to functional limitations, which in turn lead to disability in ADL [22]. In fact, multimorbidity has been found to be associated with physical impairments and functional limitation [23]. This could explain why some people are affected by multimorbidity, but not yet by disability in ADL.

When we analysed disability prevalence according to specific pairs of chronic conditions, we found very large variations. CVD combinations were associated with the lowest prevalence of disability, whereas certain mental (dementia, depression), cerebrovascular and musculoskeletal (hip fracture) diseases were associated with the highest prevalence of disability. In particular, a high prevalence of disability was found in persons affected by pairs of diseases in which one of the two diseases was dementia. As the occurrence of dementia increases with increasing age [24], this finding could explain the elevated prevalence of disability always found in studies on the oldest-old [25].

An interesting finding of this study is the effect of the aggregation amongst some specific disease pairs. The majority of these pairs consisted of the same combinations of four specific diseases (dementia, depression, CVD, and hip fracture), as well as the pair of CVD and hypertension. Moreover, having both diseases amongst these specific pairs appears to be associated with a much higher risk of disability, compared with having just one of either of the diseases in the pair. Unfortunately, due to the limitations of sample size, when examining each specific pair of diseases stratified into these four categories, the CIs are wide, and thus it cannot be concluded from these data that having both diseases is significantly more risky than having either one alone. However, the data are suggestive of this conclusion, particularly for the non-dementia disease in the pair. Indeed, for the dementia and CVD pair, it can be concluded that in our data, having dementia in addition to CVD significantly increases the relative odds of disability over having CVD but not having dementia. The same conclusion can also be reached for depression in the dementia and depression pair. For the specific disease pair of depression and CVD, the data are particularly suggestive of a synergistic effect in the risk of disability when having both diseases, compared with the risk of having either one individually. Despite the fact that we cannot draw any conclusion on a causal relationship due to the cross-sectional design of the study, depression is often observed after stroke and it can influence functional recovery [26].

Prevention of specific chronic diseases and disability is the major goal of public health professionals. In terms of disability prevention, primary prevention should avert the onset of diseases and secondary prevention should focus on the early detection and treatment of those conditions. Finally, tertiary prevention aims to reduce the impact of disability, such as institutionalization. Nowadays, all three steps are fundamental, but often cannot be applied. If we think, for example, of dementia, the most disabling chronic disease amongst the elderly [27], we can easily realize that both primary and secondary prevention are still limited. On the other hand, other chronic diseases that often occur with dementia and which increase their effect on disability, such as hip fracture and depression, could be more easily prevented or treated earlier, even at a very advanced age. Finally, three specific pairs of CVDs were found to be significantly associated with a decreased relative odds of disability, which can be interpreted as a survival bias in this very old population. By this, we mean the most severe CVDs may have already caused mortality prior to the start of this study (a competing risk to study participation), and the CVDs present in our study population thus may represent the ‘healthy survivors’ or less severe disease (less likely to be associated with any disability). Indeed, we could hypothesize a survival selection bias for many highly lethal diseases, such as HF. Unfortunately, the competing risk of early mortality against participation in late-life studies is a universal challenge faced in the field of geriatric epidemiology.

The identification of diseases or groups of diseases with the highest association with disability is important for prevention and for planning resource distribution. In both developed and developing countries, the funding of care for disabled elderly people is challenging to healthcare systems. It has been shown that medical spending in the old population is more related to disability than longevity [7, 28]. This is particularly true for people with disabilities in ADL as they need constant care and support by formal and informal caregivers, and are at high risk of being institutionalized [25]. New strategies for preventing disability may target not only single conditions but also specific disease combinations, such as aiming to prevent progression in severity of one or both of the diseases in order to reduce their effect on disability. Another strategy could be the prevention of a second disease when a specific one is already present, or ideally of disease clustering – but further prospective studies are needed in order to determine the causal pathways in disease aggregation.

It is difficult to compare the findings of this study with others as very few data are available in the literature on disease clustering and disability in the very old. Most previous studies have analysed the association of single chronic diseases with disability, yet have found similar results, such as a study on an oldest-old Chinese population showing that fractures and stroke were associated with poorer functional abilities, whereas hypertension had a better functional status [29]. Verbrugge and colleagues showed that when arthritis occurs in the presence of other specific comorbid conditions, there is an increased effect on disability [30]. Fried and colleagues demonstrated that a substantial amount of the contribution of disease to disability may derive from the interaction of specific comorbid diseases [7], calling for further studies in order to confirm their results. Recently, data from the Cardiovascular Health Study on four chronic diseases, showed a synergistic effect on functional disability between HF and depression, HF and chronic obstructive pulmonary disease, depression and arthritis, and depression and cognitive impairment [31]. In our study, the effect of cognitive impairment was studied using the diagnosis of dementia, which has a well-known and strong association with disability [27]. Indeed, the association of dementia together with either hip fracture, CVD, or depression did appear to be higher than dementia alone, but we have to be cautious in our conclusions due to the wide overlapping CIs arising from the small number of people affected by these disease pairs.

## Limitations

First, the generalizability of the study is limited to other settings similar to that of this cohort: relatively highly educated elderly living in urban areas in Western countries. Second, we have analysed only pairs of diseases, but higher-order combinations of diseases probably interact in more complicated ways and have different effects on disability. In terms of the application of these findings, it needs to be noted that the disability measure used here reflects severe disability and a high need for support with the most basic ADL. Indeed, multimorbidity is likely to also be associated with earlier steps in the process of disablement (for instance, the model put forward by Nagi) in terms of physical impairments and functional limitations before progressing to the disablement measured in this study [32]. Finally, a cross-sectional analysis means that no temporal relationship can be assumed from these findings: specifically, it cannot be determined that disease onset occurred prior to the onset of disability. However, it is plausible that most of the diseases did have an earlier onset to the disability, with the exception of depression and hip fracture, which may also arise subsequent to disablement. Unfortunately, despite the fact that the Kungsholmen Project has a longitudinal design, the small number of participants that have specific pairs of diseases does not allow sufficient statistical power to evaluate the longitudinal association between pairs of diseases and disability development during time. On the other hand, the present study has the advantage of being population-based, including subjects both living at home and in institutions. In addition, different sources of medical diagnoses were employed, including direct clinical examination, which reduced potential ascertainment biases that often affect the accuracy of the assessment of health status in the elderly, as well as the direct examination concerning ADLs.

## Conclusion

The results of this population-based study suggest that specific pairs of diseases are much more highly associated with disability than others, particularly those coupled together with dementia. This knowledge may improve prevention of disablement and planning of resource distribution.

## Figures and Tables

**Table 1:** Sociodemographic characteristics of the study population by functional status at baseline (activities of daily living): number (*n*) and percent distribution.

Characteristics	All (*n*=1,084)	No disability (*n*=890)	Disability (*n*=194)
Age (years, mean ± SD)	84.5±4.5	83.9±4.1	87.2±5.0^a^
Female (%)	837 (77.2)	676 (76.0)	161 (83.0)^b^
Education^c^ (% ≤7years)	550 (51.1)	443 (49.9)	107 (56.3)
Widowed (%)	582 (53.7)	464 (52.1)	118 (60.8)^b^
Living at home (%)	892 (82.3)	821 (92.2)	71 (36.6)^a^

^a^*p*<0.001; ^b^*p*<0.05; ^c^Data on educational background were missing for seven subjects (*n*=887 no disability; *n*=190 disability).

**Table 2:** Disability in activities of daily living according to number (*n*) and prevalence (P) of diseases and organ systems per 100 persons.

	All (*n*=1,084)		Disability (*n*=194)	
	*n*	P	P
Number of chronic diseases			
0	161	14.9	4.3
1	328	30.3	16.8
2	262	24.2	20.6
3	190	17.5	20.0
4+	143	13.2	28.0
*p* for trend			<0.001
Organ systems			
Cardiovascular	556	51.3	11.9
Malignancy	56	5.2	12.5
Respiratory	59	5.4	13.6
Blood	145	13.4	14.5
Musculoskeletal	75	6.9	17.3
Endocrine	126	11.6	18.3
Neurosensory	232	21.4	19.4
Cerebrovascular	81	7.5	42.0
Mental	277	25.6	50.9

**Table 3:** Odds ratio (OR) and 95% confidence intervals (CI) testing the association of each different pairs of diseases with disability. Models adjusted for age, sex, and education.

	*n*	OR	95% CI
HF and CHD	60	0.32	0.11–0.91
AF and hypertension	60	0.24	0.08–0.68
CHD and hypertension	83	0.29	0.12–0.69
HF and AF	42	0.44	0.15–1.29
Diabetes and hypertension	28	0.83	0.28–2.49
HF and hypertension	165	0.70	0.4–1.1
Deafness and visual impairment	24	0.55	0.19–1.58
HF and diabetes	20	2.65	0.97–7.24
CVD and hypertension	38	2.31	1.10–4.87
Depression and hip fracture	6	2.76	0.51–14.9
Depression and CVD	12	14.59	4.00–53.17
Dementia and depression	30	29.39	12.4–69.59
Dementia and CVD	30	39.83	16.53–95.96
Dementia and hip fracture	17	56.36	15.28–207.81

AF, atrial fibrillation; CHD, coronary heart disease; CVD, cerebrovascular disease; HF, heart failure; *n*, number of participants affected with both diseases. Total sample size for each model was 1,077. The reference group for each model is persons not having either of the two diseases present in the specific pair.

**Table 4:** Odds ratio (OR) and 95% confidence intervals (CI) of the pairs of diseases associated most highly with increased relative odds of disability. Models adjusted for age, sex, education.

	*n*	OR	95% CI
Dementia and hip fracture	17	56.3	15.3–207.8
Only dementia, no hip fracture	204	17.0	11.2–25.9
No dementia, only hip fracture	23	12.0	4.9–29.3
No dementia, no hip fracture	840	Ref	Ref
Dementia and CVD	30	39.8	16.5–96.0
Only dementia, no CVD	191	15.3	10.0–23.4
No dementia, only CVD	51	4.4	2.1–9.0
No dementia, no CVD	812	Ref	Ref
Dementia and depression	31	29.4	12.4–69.6
Only dementia, no depression	190	15.6	10.2–23.9
No dementia, only depression	56	3.1	1.5–6.4
No dementia, no depression	807	Ref	Ref
Depression and CVD	12	14.6	4.0–53.2
Only depression, no CVD	75	2.4	1.4–4.3
No depression, only CVD	69	3.9	2.2–6.7
No depression, no CVD	928	Ref	Ref

CVD, cerebrovascular disease; *n*, number of participants affected; Ref, reference group.
